# Variable myocardial interstitial expansion by T1 mapping within LGE area in infarction and hypertrophic cardiomyopathy

**DOI:** 10.1186/1532-429X-15-S1-P168

**Published:** 2013-01-30

**Authors:** Viviana Maestrini, Daniel Sado, Steven K White, Marianna Fontana, Sanjay M Banypersad, Thomas A Treibel, Derek J Hausenloy, James Moon

**Affiliations:** 1 The Heart Hospital, London, UK; 2 The Hatter Cardiovascular Institute, University College London Hospitals NHS Trust, London, UK

## Background

Late gadolinium enhancement (LGE) identifies focal myocardial fibrosis. However it misses diffuse fibrosis and makes all "above threshold" areas of ECV expansion appear as LGE. We hypothesized that LGE area in different cardiac diseases would have varying degrees of interstitial expansion and consequently have different ECV values.

## Methods

75 patients were prospectively enrolled: "Chronic MI": 6 months after myocardial infarction (n=25); "HCM" - hypertrophic cardiomyopathy (n=25), and healthy volunteers (n=25). Patients with HCM and no LGE were excluded. As the conditions for dynamic contrast equilibrium are not reached in infarction, equilibrium contrast was performed. The T1 mapping sequence was ShMOLLI. The contrast agent was Gadoterate meglumine (Dotarem) at 0.1mmol/Kg (bolus) plus infusion at 15minutes at 0.0011 mmol/kg/min. CMR was at 1.5T (Siemens Avanto). Regions of interest were drawn in the LV blood pool, the region of LGE and in the remote myocardium (which was a non-hypertrophied area in the HCM patients) to generate ECV values calibrated to hematocrit.

## Results

**Figure 1:**
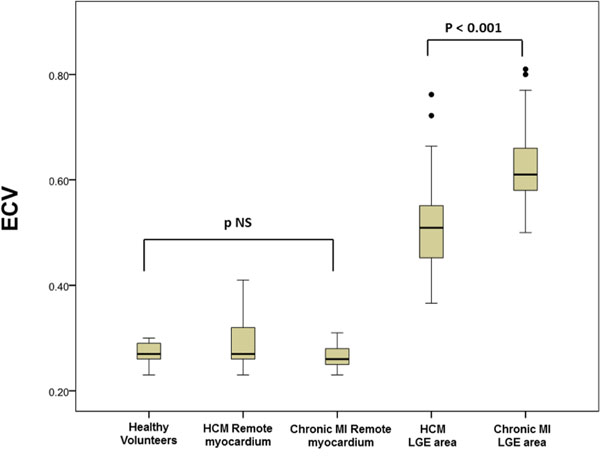
HCM vs chronic MI-ECV in LGE area and in remote myocardium

LGE in HCM had a lower ECV than LGE in chronic MI (0.52±0.10 vs 0.63±0.09; p<0.001 - Figure 1). No significant difference was detected between remote myocardial ECV in HCM and chronic MI patients and healthy volunteers (respectively 0.29±0.04, 0.27±0.02, 0.27±0.02; p=NS).

## Conclusions

Not all LGE is the same, with the ECV in LGE areas of HCM being lower than that of chronic infarction.

## Funding

None

